# Cord Serum Concentrations of Perfluorooctane Sulfonate (PFOS) and Perfluorooctanoate (PFOA) in Relation to Weight and Size at Birth

**DOI:** 10.1289/ehp.10334

**Published:** 2007-07-31

**Authors:** Benjamin J. Apelberg, Frank R. Witter, Julie B. Herbstman, Antonia M. Calafat, Rolf U. Halden, Larry L. Needham, Lynn R. Goldman

**Affiliations:** 1 Department of Epidemiology, Johns Hopkins Bloomberg School of Public Health, Baltimore, Maryland, USA; 2 Department of Gynecology and Obstetrics, Johns Hopkins University School of Medicine, Baltimore, Maryland, USA; 3 Columbia Children’s Center for Environmental Health, Columbia Mailman School of Public Health, New York, New York, USA; 4 Division of Laboratory Sciences, National Center for Environmental Health, Centers for Disease Control and Prevention, Atlanta, Georgia, USA; 5 Department of Environmental Health Sciences, Johns Hopkins Bloomberg School of Public Health, Baltimore, Maryland, USA

**Keywords:** Birth weight, cord blood, epidemiology, fetal exposure, fetal growth, gestational age, head circumference, human, length, perfluorooctane sulfonate, perfluorooctanoate, polyfluoroalkyl compounds, ponderal index

## Abstract

**Background:**

Recent studies have reported developmental toxicity among rodents dosed with perfluorooctane sulfonate (PFOS) and perfluorooctanoate (PFOA).

**Objectives:**

We examined the relationship between concentrations of PFOS and PFOA in cord serum (surrogates for *in utero* exposures) and gestational age, birth weight, and birth size in humans.

**Methods:**

We conducted a hospital-based cross-sectional epidemiologic study of singleton deliveries in Baltimore, Maryland. Cord serum samples (*n* = 293) were analyzed for PFOS and PFOA by online solid-phase extraction, coupled with reversed-phase high-performance liquid chromatography–isotope dilution tandem mass spectrometry. Maternal characteristics and anthropometric measures were obtained from medical charts.

**Results:**

After adjusting for potential confounders, both PFOS and PFOA were negatively associated with birth weight [per ln-unit: β = −69 g, 95% confidence interval (CI), −149 to 10 for PFOS; β = −104 g, 95% CI, −213 to 5 for PFOA], ponderal index (per ln-unit: β = −0.074 g/cm^3^ × 100, 95% CI, −0.123 to −0.025 for PFOS; β = −0.070 g/cm^3^ × 100, 95% CI, −0.138 to −0.001 for PFOA), and head circumference (per ln-unit: β = −0.32 cm, 95% CI, −0.56 to −0.07 for PFOS; β = −0.41 cm, 95% CI, −0.76 to −0.07 for PFOA). No associations were observed between either PFOS or PFOA concentrations and newborn length or gestational age. All associations were independent of cord serum lipid concentrations.

**Conclusions:**

Despite relatively low cord serum concentrations, we observed small negative associations between both PFOS and PFOA concentrations and birth weight and size. Future studies should attempt to replicate these findings in other populations.

Polyfluoroalkyl compounds (PFCs) comprise a class of man-made, fluorinated organic compounds that have been used in a variety of consumer and industrial applications. Although these have been produced for many years, only recently have reports surfaced suggesting widespread exposure in wildlife and humans (Butenhoff et al. 2006; Calafat et al. 2007; Giesy and Kannan 2001; Houde et al. 2006; Kannan et al. 2004). Two of the most widely detected and studied compounds in this class are perfluorooctane sulfonate (PFOS) and perfluorooctanoate (PFOA). PFOS and related compounds (polyfluorinated sulfonamides) are surfactants used in applications ranging from oil and water repellents for fabrics, apparel, carpets, and paper coatings to specialty chemical applications such as insecticides and fire fighting foams (3M Company 1999). PFOA and its salts are used as chemical intermediates and processing aids in the production of fluoropolymers and fluoroelastomers.

Both PFOS and PFOA have shown the potential for developmental toxicity in animal studies. PFOS has been shown, in rats and mice, to induce developmental and reproductive effects, such as reduced birth weight, decreased gestational length, structural defects, developmental delays, and increased neonatal mortality (Fuentes et al. 2006; Grasty et al. 2003; Lau et al. 2003; Luebker et al. 2005a, 2005b; Thibodeaux et al. 2003). Recent studies have also reported developmental toxicity from PFOA in rodents, including pregnancy loss, reduced fetal weight, reduced postnatal survival, and delays in postnatal growth and development in offspring (Butenhoff et al. 2004; Lau et al. 2004, 2006). However, such studies of PFOS and PFOA have been conducted using doses that produce serum concentrations much higher than general population human exposures. There are limited epidemiologic data on the potential impacts of PFC exposure on fetal growth and development. However, one recent occupational study found no association between employment in jobs with high exposure to PFOS before the end of pregnancy and maternally reported birth weight (Grice et al. 2007).

PFOS and PFOA have also been shown to cause reductions in serum cholesterol and/or triglycerides in several animal species (Haughom and Spydevold 1992; Seacat et al. 2002, 2003; Thibodeaux et al. 2003). Conversely, a few cross-sectional occupational studies conducted among fluorochemical production employees have reported positive relationships between PFOS and/or PFOA concentrations and serum lipid levels (Gilliland and Mandel 1996; Olsen et al. 1999, 2003). The fetus is likely to be sensitive to the availability of cholesterol and triglycerides, which support cellular growth, differentiation, and adipose accumulation (Woollett 2001). Disruptions to normal fetal growth and development have been associated with effects across the lifespan, including adverse neonatal and childhood outcomes (Hofman et al. 1997; Kramer et al. 1990) and metabolic diseases in adulthood (Barker 2006).

In a previous report, we documented factors associated with cord serum concentrations of PFOS and PFOA in a population of newborn deliveries known as the Baltimore THREE study (Apelberg et al. 2007). In this study, we examined the relationship between these concentrations and gestational age, birth weight, and measures of birth size, including head circumference, length, and ponderal index (a measure of body mass at birth).

## Materials and Methods

### Subjects

We conducted a cross-sectional study (the Baltimore THREE Study) of newborn deliveries at the Johns Hopkins Hospital in Baltimore, Maryland. This study received approval from the Johns Hopkins Medicine Institutional Review Board and received a waiver from the Health Insurance Portability and Accountability Act. The study required the collection only of specimens that otherwise would have been discarded and information from medical records that were available to hospital personnel. There was no requirement for informed consent due to the anonymization of all samples and data. Members of a community advisory committee, who were selected for their specific knowledge, expertise, and focus on important child health concerns in Maryland, had the opportunity to learn about and comment on this study before it was conducted. Between 26 November 2004 and 16 March 2005, all singleton, live birth deliveries occurring in the labor and delivery suite at the hospital were eligible for participation in the study. We excluded women who gave birth to multiple children or women who delivered a single child but had an initial twin gestation with fetal loss at ≥ 20 weeks. Newborns with major congenital anomalies likely to affect fetal growth were excluded as well.

Over the course of the study period, 609 live births occurred at the Johns Hopkins Hospital, of which 597 were singleton births. We obtained cord blood specimens from 341 of these, of which 42 had insufficient volume for laboratory analyses and were excluded from this study. Additionally, six births were excluded due to known major congenital anomalies (*n* = 5) or an initial twin gestation (*n* = 1), leaving a total of 293 study participants. We conducted a brief survey of hospital personnel to understand the major reasons for missed specimen collection. The most common explanations included complications during delivery, premature birth and/or small size of the infant resulting in small quantity of available cord blood, and logistical factors such as understaffing. The babies who were not included had somewhat lower gestational ages and birth weights. In addition, factors associated with insufficient blood volumes collected were preterm birth, low birth weight, being first born, and younger age of mother.

Detailed methods for the collection and analysis of cord blood samples are described in a prior publication (Apelberg et al. 2007). Briefly, samples were collected from the umbilical cord vein by hospital personnel using a sterile technique immediately after a singleton birth delivery (Witter et al. 2001). Within 3 hr, refrigerated specimens were centrifuged and serum was stored at −80°C in 2-mL polypropylene cryovials, previously pre-screened and shown to be free of PFC contaminants. Specimens were transferred on dry ice to the Centers for Disease Control and Prevention (CDC) for laboratory analyses.

### Medical records

We abstracted maternal and infant characteristics from clinical databases maintained by the hospital. Maternal information was abstracted by two study investigators concurrently (B.J.A., J.B.H.), and a random 10% sample was verified by two others (L.R.G., F.R.W.). Additional information was obtained from forms filled out by the nursing staff at the time of delivery. Age, race, education, and parity were based on self-report. Body mass index (BMI) was calculated from reported prepregnancy weight and height. Gestational age was based on the best obstetric estimate. Infant sex was abstracted from the mother’s medical record and confirmed with the infant record. Information on maternal health conditions was abstracted from the medical record. Maternal smoking status at birth was defined using a combination of the maternal medical record and cord serum cotinine concentrations. Cotinine concentrations > 10 ng/mL were categorized as exposure to maternal active smoking. If the clinical record indicated that the mother reported smoking at any time during pregnancy, she was considered an active smoker regardless of the cotinine concentration in cord serum.

Infant anthropometric measures were abstracted from the infant medical record. Birth weight in grams obtained from the maternal record was confirmed with the infant record. Head circumference and length in centimeters were abstracted from the infant record. Ponderal index was calculated as the ratio of birth weight in grams to length in centimeters cubed, multiplied by 100 (*birth-weight/length^3^* × *100*). We examined the relationships among gestational age, birth weight, length, and head circumference to identify outlying values, which were verified using the infant and maternal record. All congenital conditions on the birth record were recorded and reviewed by a clinician blinded to exposure status to determine which subjects had major malformations and should be excluded from this study. Because only singleton births were eligible for this study, we retrospectively excluded one birth, which, unknown to us at the time of sample collection, was from a twin gestation with demise of the other twin at 20 weeks.

### Laboratory analysis

#### Polyfluoroalkyl compounds

We analyzed cord serum samples for PFOS, PFOA, and nine other PFCs using a modification of the method of Kuklenyik et al. (2005), described in detail previously (Apelberg et al. 2007; Kuklenyik et al. 2005). This analytical methodology has been used to measure PFCs in large-scale surveys, including the National Health and Nutrition Examination Survey (NHANES) (Calafat et al. 2007). Briefly, without protein precipitation, the analytes in one aliquot of 100 μL of serum were preconcentrated using automated solid phase extraction, chromatographically resolved by reversed-phase high performance liquid chromatography (HPLC), and detected by negative-ion TurboIonSpray ionization-isotope dilution-tandem mass spectrometry (MS/MS). For quantification, we used ^18^O_2_-PFOS and ^18^O_2_-PFOSA (RTI International, Research Triangle Park, NC) and ^13^C_2_-PFOA provided by Dupont Co. (Wilmington, DE). The limit of detection (LOD), calculated as 3S_0_, where S_0_ is the standard deviation as the concentration approaches zero (Taylor 1987), was 0.2 ng/mL for both PFOS and PFOA (Kuklenyik et al. 2005); the limit of quantitation was three times the LOD. In these cord serum samples, the precursor/product ion *m/z* transition used for the quantification of PFOS (499/99) had an interference. Therefore, PFOS concentrations were calculated using another transition (499/130), normally used to confirm the presence of PFOS. The standard accuracies (93–118%) were obtained at three spike levels [LOD, 1.25 ng/mL (6.6 ng/mL for PFOS) and 12.5 ng/mL (50 ng/mL for PFOS)] (Kuklenyik et al. 2005). Quality control (QC) materials [low-concentration (~ 3 ng/mL to ~ 9 ng/mL) and high-concentration (~ 10 ng/mL to ~ 30 ng/mL)], prepared from a base calf serum pool, and reagent blank samples were included in each analytical batch along with the unknown samples and evaluated according to standard statistical probability rules (Kuklenyik et al. 2005). Under the experimental conditions described above, the coefficients of variation of repeated measurements of the QC materials, which reflect the inter-batch precision within a period of 9 months, were 12.9–20.6% (PFOS) and 10.7–11.6% (PFOA). All laboratory analyses were conducted by investigators blinded to the characteristics of study subjects.

##### Cotinine

Concentrations of cotinine in serum were determined using a method described by Bernert et al. (1997). It employs HPLC coupled with atmospheric pressure chemical ionization MS/MS to measure serum cotinine concentrations with high accuracy and sensitivity (LOD = 0.015 ng/mL). This method (Bernert et al. 1997) has been used to assess exposure to environmental tobacco smoke in NHANES and other large-scale surveys.

##### Lipids

Serum lipid concentration analyses were conducted using commercially available test kits from Roche Diagnostics Corp. (Indianapolis, IN) for the quantitative determination of total triglycerides (product no. 011002803-0600) and total cholesterol (product no. 011573303-0600). Final determinations were made on a Hitachi 912 Chemistry Analyzer (Hitachi, Tokyo, Japan).

### Statistical analysis

We used descriptive statistics appropriate for right-skewed data to characterize PFOS and PFOA concentrations and the Kruskall-Wallis test to compare median concentrations across demographic characteristics. Spearman rank correlation was used to estimate the correlation between cord serum concentrations of the two compounds. Because PFOS and PFOA concentrations were skewed to the right, all statistical tests requiring assumptions of normality were conducted on natural log-transformed concentrations. Natural log-transformed concentrations were also used as independent variables in regression analysis to minimize the potential influence of extreme values on the regression coefficients. Further, natural log-transformed concentrations provided a better fit than untransformed data in most regression models, based on the Akaike Information Criterion (Akaike 1973) and the model *R*^2^. Concentrations below the LOD for PFOS, PFOA, and cotinine were set to the LOD divided by the square root of two for all analyses (Hornung and Reed 1990).

We conducted univariate and multivariate linear regression analyses to examine associations between PFOS or PFOA and gestational age, birth weight, head circumference, length, and ponderal index. Key determinants of gestational age included in regression models were smoking status, age, race, prepregnancy BMI, previous preterm birth, diabetes, and hypertension. Key determinants of birth weight and birth size included in the primary adjusted models were smoking status, age, gestational age, race, prepregnancy BMI, net weight gain during pregnancy (weight gain minus birth weight), height, parity, infant sex, diabetes, and hypertension. Diabetes was defined to include subjects with preexisting or gestational diabetes, assuming that both conditions would result in increased fetal weight for age. Similarly, hypertension was defined to include subjects with preeclampsia, pregnancy-induced hypertension, and chronic hypertension, which were expected to result in decreased weight for age. For consistency, the same set of covariates was included in the primary regression model for each end point other than gestational age, except for delivery mode [vaginal versus caesarian section (C-section)], which was included as a predictor of head circumference. We also explored the possibility of an interaction between delivery mode and cord PFOS or PFOA concentrations on head circumference. Based on the empirical evidence, a quadratic term for maternal age was included in regressions of gestational age, birth weight, head circumference, and ponderal index, but not for length. A quadratic term for gestational age was included in regressions of head circumference only. All other terms were included in the models as linear or categorical (indicator) variables. We considered maternal education level as a measure of socioeconomic status, but it was not included in the final models because it did not materially change the coefficient estimates. We also conducted regressions before and after controlling for total lipids, total cholesterol, and triglycerides to examine the possible role of serum lipids as a mediator of any relationships between PFOS or PFOA concentrations and birth weight and size.

A small number (< 4%) of participants were missing data on prepregnancy weight, height, and/or net weight gain during pregnancy, which are important predictors of birth weight and size. We imputed the missing data with the median value of height, weight, and/or weight gain. As a sensitivity analysis, we examined the impact of imputing BMI or weight gain with extreme values and found no material effect on the regression coefficients for PFOS or PFOA.

Regression diagnostics were conducted for all models, including examination of fit, heteroskedasticity, and influence. Statistical analyses were performed using STATA version 8.0 (StataCorp., College Station, TX).

## Results

PFOA was detected in 100% of cord blood serum samples, and PFOS was detected in > 99% of samples. The median PFOA concentration was 1.6 ng/mL (range, 0.3 to 7.1 ng/mL) and the median PFOS concentration was 5 ng/mL [range, < LOD (0.2) to 34.8 ng/mL]. Concentrations of PFOS and PFOA in cord serum were highly correlated (Spearman rank correlation coefficient, 0.58; *p* < 0.01).

Table 1 shows the characteristics of the study population stratified by median (and interquartile range) cord PFOS and PFOA concentrations. Statistically significant differences (*p* < 0.05) in median PFOS concentrations were observed by race, smoking status, and hypertension, and differences in median PFOA concentrations were observed by BMI category, parity, and infant sex. The distribution of fetal growth indices are shown in Table 2.

Table 3 shows univariate and multivariate regression model results of gestational age, birth weight, length, head circumference, and ponderal index on natural log-transformed PFOS and PFOA concentrations. The coefficients from these regression models are equivalent to a change in the end point associated with a 2.7-fold increase in PFOS or PFOA. To present these findings on the arithmetic scale for PFOS and PFOA, we also converted the adjusted coefficients into the estimated difference in each end point associated with an increase from the 25th to 75th percentile of concentration (Table 3). This corresponds to an increase in concentration from 3.4 to 7.9 ng/mL for PFOS and from 1.2 to 2.1 ng/mL for PFOA. Multivariate models of birth weight and size are presented both adjusted for gestational age only and fully adjusted as described above.

**Figure 1:**
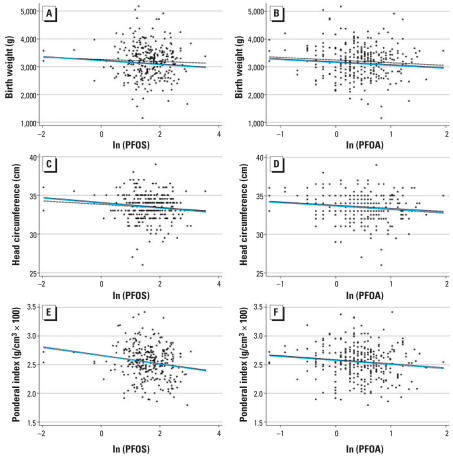
Birth weight (*A*, *B*), head circumference (*C*, *D*), and ponderal index (*E*, *F*) versus ln(PFOS) and ln(PFOA) concentrations, before and after adjustment for potential confounders. The black dotted lines denote the predicted fit from a simple linear regression model. The blue solid lines denote the predicted fit from the fully adjusted multivariate regression model. Corresponding regression coefficients are presented in Table 3.

A non-statistically significant positive association was observed between PFOS and PFOA and gestational age, which diminished with adjustment for key predictors of gestational age. After adjusting for gestational age, both chemicals were negatively associated with birth weight (*p* < 0.05). Further adjustment for additional covariates resulted in an attenuation of this association. In the fully adjusted model, a ln-unit increase in cord concentration was associated with a decrease in mean birth weight of 69 g [95% confidence interval (CI), −149 to 10) for PFOS and 104 g (95% CI, −213 to 5) for PFOA. For head circumference, a negative association was observed with PFOS in multivariate analyses. Under the fully adjusted model, an increase in one ln-unit of PFOS was associated with a decrease in mean head circumference of 0.32 cm (95% CI, −0.56 to −0.07). For PFOA, a negative association was observed in both univariate and multivariate analyses. In the fully adjusted model, a ln-unit increase in PFOA was associated with a decrease in mean head circumference of 0.41 cm (95% CI, −0.76 to −0.07). The association between PFOA and head circumference was also consistent with a curvilinear relationship on the log scale, which was confirmed by inclusion of a quadratic term (*p* < 0.05) in the model (data not shown). Negative associations were observed for both PFOS and PFOA with ponderal index in univariate and multivariate models. After full adjustment, a ln-unit increase in PFOS concentration was associated with a decrease in average ponderal index (g/cm^3^ × 100) of 0.074 (95% CI, −0.123 to −0.025). An increase in PFOA equal to one ln-unit was associated with a decrease in mean ponderal index (g/cm^3^ × 100) of 0.070 (95% CI, −0.138 to −0.001). In contrast, neither PFOS nor PFOA was significantly associated with newborn length in univariate or multivariate models (Table 3). The predicted values from selected regression models are plotted across the full range of ln(PFOS) and ln(PFOA) concentrations in Figure 1.

We examined the relationships between PFOS and PFOA concentrations with serum total cholesterol, triglycerides, and total lipids and found no evidence of an association between chemical exposure and any of these biomarkers. Consequently, adjusting for these measures had no impact on the associations observed between PFOS or PFOA and the end points under study [see Supplemental Material (online at http://www.ehponline.org/docs/2007/10334/suppl.pdf)].

As expected, delivery mode was associated with head circumference, with babies born by C-section having larger head circumferences, on average, compared with vaginal deliveries, after adjusting for potential confounders. Because of the potential for increased measurement error due to head molding in vaginal deliveries, we included an interaction term between PFOS and PFOA with delivery mode, which was statistically significant (*p* < 0.05). Surprisingly, the negative association was restricted to vaginal births. Among vaginal births, both PFOS and PFOA were negatively associated with head circumference [−0.46 cm per ln-unit PFOS (95% CI −0.73 to −0.19); −0.62 cm per ln-unit PFOA (95% CI −1.0 to −0.23)]. Among C-sections, there was a nonsignificant positive association between both PFOS and PFOA and head circumference. To further explore this issue, we evaluated whether this statistical interaction was also present for birth weight and ponderal index (data not shown). For birth weight, some evidence of an interaction in the same direction was observed with PFOA (*p* < 0.05) and PFOS (*p* = 0.08). No evidence of an interaction was observed for ponderal index.

## Discussion

In a previous study, we documented the magnitude and determinants of fetal cord serum concentrations of PFOS and PFOA among deliveries occurring at a hospital in Baltimore, Maryland (Apelberg et al. 2007). In this study, we examined the relationship between these concentrations and gestational age, birth weight, and birth size. Our results showed negative associations between both PFOS and PFOA concentrations and birth weight and ponderal index, after adjusting for potential confounders. We also observed a negative association between both PFOS and PFOA concentrations and head circumference. For reasons unknown, the negative associations with birth weight and head circumference were observed only among vaginal deliveries. However, these comprised most of our population (77.8%). This finding could be attributed to chance or could be associated with underlying complications that led to a delivery by C-section in our population. By contrast, no significant associations were observed between either PFOS or PFOA concentrations and newborn length or gestational age. Adjusting for gestational age had the greatest impact on the association between PFOS and PFOA concentrations and birth weight and size, which tended to strengthen the associations observed.

Head circumference has been used as a measure of brain growth and development (Lindley et al. 1999). Future studies will be needed to evaluate whether this finding can be confirmed and, if so, whether there are associated alterations in neurodevelopment. Ponderal index has been used by clinicians and epidemiologists as a measure of thinness at birth and an indicator of disproportionate or asymmetric growth restriction. Fetal weight and soft tissue mass increase dramatically in the third trimester and the body becomes more proportional. It has been hypothesized that early insults during gestation result in symmetric growth restriction and later insults result in asymmetric growth restriction, but some empirical evidence conflicts with this notion about the timing of fetal growth disruption (Cunningham et al. 2001; Vik et al. 1997). Kramer et al. (1989) showed that disproportionality increased with increasing severity of growth restriction, suggesting the presence of a continuum of fetal growth restriction, rather than two distinct patterns. Regardless, several studies have shown associations between low ponderal index and risk of adverse perinatal outcomes (Cheung et al. 2002; Fay et al. 1991; Nieto et al. 1998; Villar et al. 1990).

The toxicology literature provides evidence of developmental effects among animals dosed with PFOS and PFOA, albeit at substantially higher levels than observed here (Butenhoff et al. 2004; Fuentes et al. 2006; Grasty et al. 2003; Lau et al. 2003, 2004, 2006; Luebker et al. 2005a, 2005b; Thibodeaux et al. 2003). For example, Luebker et al. (2005b) estimated a BMDL_5_ (lower bound of the benchmark dose associated with a 5% change in response) for PFOS and birth weight in rats of 0.39 mg/kg/day, equivalent to a rat fetal serum concentration of about 34,000 ng/mL. Butenhoff et al. (2004) reported BMDL_10_s for several postnatal developmental end points for PFOA in rats, ranging from 22 to 44 mg/kg/day, equivalent to rat fetal serum concentrations from 29,000 to 59,000 ng/mL. Thus, the serum concentrations of these compounds associated with developmental effects in rats are several orders of magnitude higher than the concentrations observed here.

Only limited epidemiologic data on PFCs and developmental outcomes exist. In one recent occupational epidemiologic study of perfluorooctanesulfonyl fluoride manufacturing workers, investigators examined the relationship between occupational PFOS exposure and self-reported medical conditions (Grice et al. 2007). Among female respondents, a pregnancy history was ascertained, including the number of previous pregnancies and the weight (by recall) of all live births. The investigators found no association between cumulative PFOS exposure, based on a simple job exposure matrix, and maternally reported birth weight. However, several key determinants of birth weight were not adjusted for—most notably, the length of gestation—and the extent of follow-up of workers who left the company is unclear (Grice et al. 2007).

Hypolipidemic effects have been among the sensitive changes observed in animals in response to PFOS and PFOA exposure (Haughom and Spydevold 1992; Seacat et al. 2002, 2003; Thibodeaux et al. 2003). In occupational studies, associations between exposure and cholesterol and triglyceride levels have been observed as well, although in the opposite direction (Gilliland and Mandel 1996; Olsen et al. 1999, 2003). It is plausible that if PFOS and PFOA were modifying lipid metabolism during pregnancy, this could be related to our observations of reduced weight for length. However, the observed associations between PFOS or PFOA and ponderal index were independent of cord serum lipid levels at birth. Future studies should examine the possible role of maternal or fetal lipid concentrations earlier in gestation on the associations observed here. Alternatively, alteration of fatty acid metabolism could have secondary nutritional impacts, such as on absorption of fat soluble vitamins. Further studies could determine whether levels of fat-soluble vitamins mediate or modify the associations between PFOS and PFOA concentrations and weight.

There are some potential limitations to this cross-sectional study, described below, which should lead to cautious interpretation of the results. We used a precise analytical method for the detection of subparts per billion PFC concentrations in human serum (Kuklenyik et al. 2005). In this study, however, measurement error for PFOS may be greater than expected based on the method performance, because a different ion transition had to be used for quantification. In addition, anthropometric measurements such as head circumference and length are likely to have a larger degree of measurement error than birth weight, due both to head molding and the potential for measurement subjectivity. However, measurement error of either the exposure or end point would be expected to bias bivariate associations to the null if the error is completely random (Brenner and Loomis 1994). Further, there is a range of normal variation for these end points in a population and small changes in weight and size at birth would not necessarily have clinical significance.

Given the relatively low serum concentrations, future studies should examine possible noncausal mechanisms that may be responsible for the associations reported here. PFOS and PFOA have an affinity for protein-binding and have been shown to accumulate in protein-rich compartments in biological systems (Jones et al. 2003; Luebker et al. 2002; Vanden Heuvel et al. 1992). Because serum protein levels may be related to nutritional status, further studies should examine the potential role protein concentrations may play in the associations observed between fetal exposure to these chemicals and fetal growth. Additionally, poor maternal plasma volume expansion has been associated with smaller size at birth (Salas et al. 1993, 2006). If, hypothetically, reduced maternal plasma volume expansion led to higher cord blood concentrations of PFOS or PFOA, this could account for the associations observed here. However, preeclampsia and pregnancy-induced hypertension are both associated with poor maternal plasma volume expansion (Salas et al. 2006), yet cord serum PFOS and PFOA concentrations were not elevated among women with these conditions (Table 1). Placental weight is also related to maternal plasma volume (Salas et al. 1993). When preeclampsia and hypertension, as well as placental weight (data not shown), were added to the regression models, the associations between PFOS and PFOA and growth outcomes did not change. Although we did not find support for this alternative explanation, future studies could examine whether plasma–volume changes during pregnancy may be responsible for the observed associations.

The use of medical records as the principal source for data on potential confounders is likely to result in some degree of misclassification. However, we observed the expected relationships between key predictors and birth outcomes [see Supplemental Material (online at http://www.ehponline.org/docs/2007/10334/suppl.pdf)], suggesting that the degree of residual confounding is likely to be small. Although we adjusted for the major known determinants of birth size and weight, it remains possible that other unmeasured factors, such as diet, may be confounding the relationships observed in this study. The maternal diet would be expected to be related to weight and size of the fetus, and it remains possible that the consumption of contaminated food or water, including the use of fast-food containers, is a pathway of exposure to PFOS and/or PFOA. To confound the relationships observed, undernutrition would have to be associated with greater PFOS or PFOA exposure. In this and in a previous publication, we reported slightly higher concentrations of both chemicals in underweight and obese mothers compared with normal weight mothers, although not all differences were statistically significant (Apelberg et al. 2007).

Finally, our study population represented a group of individuals with more risk factors for adverse birth outcomes than the United States as a whole. Compared with national estimates, the subjects in our population were more likely to be black, teenagers, unmarried, and cigarette smokers (Hoyert et al. 2006). This is perhaps not surprising given the location of the hospital in an urban and economically disadvantaged community. Although not quantified, these subjects may also have higher rates of other risk factors for poor outcomes, such as substance abuse and infections. It is not clear what, if any, impact the presence of concomitant risk factors would have on the results reported here, but future studies should be conducted in other settings to confirm these findings.

In summary, we observed a small negative association between PFOS and PFOA concentrations in cord blood and birth weight, ponderal index, and head circumference. Although lipid metabolism alterations have been among the more sensitive effects observed in animal and human data, the associations observed here were independent of cord serum lipid levels. We suggest cautious interpretation of this study until the findings can be replicated in other populations.

## Figures and Tables

**Table 1:** Distribution of study population characteristics by PFOS and PFOA concentrations.

	Median (IQR)		
Characteristic	No. (%)	PFOS (ng/mL)	PFOA (ng/mL)
Maternal age (years)			
< 18	24 (8.2)	5.0 (3.3–7.5)	1.4 (1.1–2.3)
18–35	246 (84.0)	5.0 (3.5–7.9)	1.6 (1.2–2.1)
> 35	23 (7.9)	4.1 (3.3–8.7)	1.6 (0.9–2.0)
Race*			
White	60 (20.5)	4.5 (3.1–7.1)	1.5 (1.0–2.2)
Asian	25 (8.5)	6.5 (3.4–12.3)	1.5 (1.1–2.6)
Black	208 (71.0)	5.2 (3.6–7.8)	1.7 (1.3–2.1)
Education			
< High school diploma	86 (29.8)	4.6 (3.3–7.1)	1.5 (1.2–1.9)
High school diploma	96 (33.2)	4.9 (3.7–7.9)	1.7 (1.3–2.2)
1–4 years college	65 (22.5)	5.7 (3.5–8.6)	1.6 (1.1–2.2)
≥ 5 years college	42 (14.5)	5.0 (3.4–9.1)	1.6 (1.2–2.4)
BMI** (kg/m^2^)			
Underweight (< 18.5)	16 (5.7)	6.0 (3.6–12.0)	1.8 (1.2–2.4)
Normal (18.5–24.9)	132 (46.6)	4.9 (3.4–7.4)	1.5 (1.1–1.9)
Overweight (25–29.9)	64 (22.6)	5.0 (3.2–8.1)	1.7 (1.2–2.3)
Obese (≥ 30)	71 (25.1)	5.0 (3.8–8.2)	1.8 (1.3–2.2)
Net weight gain (lbs)			
< 21.8	142 (49.8)	4.9 (3.4–6.8)	1.6 (1.2–2.1)
≥ 21.8	143 (50.2)	5.4 (3.5–8.9)	1.7 (1.2–2.2)
Maternal height (inches)			
< 64	111 (38.4)	5.0 (3.5–8.2)	1.7 (1.2–2.2)
≥ 64	178 (61.6)	4.9 (3.4–7.6)	1.6 (1.2–2.1)
Primiparous**			
Yes	122 (41.6)	5.4 (3.4–8.3)	1.7 (1.3–2.4)
No	171 (58.4)	4.6 (3.4–7.4)	1.5 (1.1–2.0)
Smoking status*			
Active	55 (18.8)	4.1 (3.3–6.2)	1.4 (1.3–1.9)
Non/passive smoker	238 (81.2)	5.2 (3.5–8.2)	1.6 (1.2–2.2)
Infant sex**			
Male	162 (55.3)	4.6 (3.2–7.7)	1.5 (1.1–1.9)
Female	131 (44.7)	5.5 (3.8–8.0)	1.8 (1.4–2.3)
Type of delivery			
Vaginal	228 (77.8)	5.0 (3.4–7.8)	1.6 (1.2–2.1)
C-section	65 (22.2)	5.0 (3.4–8.4)	1.7 (1.2–2.5)
Hypertension* (preeclampsia, pregnancy induced, and preexisting)			
Yes	33 (11.3)	4.0 (2.7–7.4)	1.3 (0.9–2.3)
No	260 (88.7)	5.2 (3.6–7.9)	1.6 (1.2–2.1)
Diabetes (gestational and preexisting)			
Yes	20 (6.8)	3.7 (2.4–8.2)	1.5 (0.7–2.2)
No	273 (93.2)	5.0 (3.5–7.9)	1.6 (1.2–2.1)
Gestational age			
Preterm	38 (13.0)	4.3 (3.1–8.0)	1.4 (1.1–1.8)
Full-term	255 (87.0)	5.0 (3.5–7.9)	1.6 (1.2–2.2)

IQR = interquartile range. Missing data were excluded from the calculation of percentages and medians. The following data were missing: 4 observations for education, 10 for BMI, 8 for net weight gain, and 4 for maternal height. Statistically significant differences (*p* < 0.05) using the Kruskall-Wallis test denoted by

* for PFOS and

** for PFOA.

**Table 2:** Distribution of study end points.

		Percentile				
End point	Mean	10th	25th	50th	75th	90th
Gestational age (days)	272	254	266	275	282	287
Birth weight (g)	3,200	2,488	2,777	3,209	3,562	3,909
Head circumference (cm)	33.5	31.0	32.5	33.5	34.5	35.5
Length (cm)	50.0	47.0	48.5	50.0	52.0	53.5
Ponderal index (g/cm^3^ × 100)	2.54	2.15	2.37	2.55	2.72	2.89

**Table 3:** Estimated change in mean gestational age, birth weight, and birth size parameters with a change in PFOS or PFOA concentrations equal to one ln-unit or from the 25th to 75th percentile.

	PFOS		PFOA	
Model	Change in end point (95% CI) per ln-unit^a^	Per increase from the 25th to 75th percentile (IQR)^b^	Change in end point (95% CI) per ln-unit^a^	Per increase from the 25th to 75th percentile (IQR)^b^
Gestational age (days)				
Univariate	2.0 (−0.4 to 4.4)	1.7 (−0.3 to 3.7)	2.4 (−0.8 to 5.7)	1.4 (−0.4 to 3.2)
Adjusted^c^	1.1 (−1.2 to 3.4)	0.9 (−1.1 to 2.9)	1.9 (−1.3 to 5.0)	1.0 (−0.7 to 2.8)
Birth weight (g)				
Univariate	−37 (−139 to 64)	−31 (−117 to 54)	−97 (−234 to 40)	−54 (−131 to 23)
Adjusted for GA	−89 (−170 to −8)*	−75 (−143 to −7)*	−161 (−270 to −52)*	−90 (−151 to −29)*
Fully adjusted^d^	−69 (−149 to 10)	−58 (−125 to 9)	−104 (−213 to 5)	−58 (−119 to 3)
Head circumference (cm)				
Univariate	−0.22 (−0.52 to 0.07)	−0.19 (−0.44 to 0.06)	−0.46 (−0.87 to −0.06)*	−0.26 (−0.48 to −0.03)*
Adjusted for GA	−0.34 (−0.59 to −0.08)*	−0.28 (−0.50 to −0.07)*	−0.54 (−0.89 to −0.19)*	−0.30 (−0.50 to −0.10)*
Fully adjusted^d^	−0.32 (−0.56 to −0.07)*	−0.27 (−0.48 to −0.06)*	−0.41 (−0.76 to −0.07)*	−0.23 (−0.42 to −0.04)*
Length (cm)				
Univariate	0.25 (−0.21 to 0.72)	0.21 (−0.18 to 0.6)	−0.06 (−0.69 to 0.57)	−0.03 (−0.39 to 0.32)
Adjusted for GA	0.05 (−0.33 to 0.44)	0.05 (−0.28 to 0.37)	−0.30 (−0.82 to 0.22)	−0.17 (−0.46 to 0.12)
Fully adjusted^d^	0.13 (−0.26 to 0.52)	0.11 (−0.22 to 0.44)	−0.10 (−0.64 to 0.44)	−0.06 (−0.36 to 0.24)
Ponderal index (g/cm^3^ × 100)				
Univariate	−0.070 (−0.119 to −0.021)*	−0.059 (−0.101 to −0.018)*	−0.074 (−0.140 to −0.007)*	−0.041 (−0.078 to −0.004)*
Adjusted for GA	−0.080 (−0.127 to −0.033)*	−0.067 (−0.107 to −0.028)*	−0.085 (−0.150 to −0.020)*	−0.048 (−0.084 to −0.011)*
Fully adjusted^d^	−0.074 (−0.123 to −0.025)*	−0.062 (−0.104 to −0.021)*	−0.070 (−0.138 to −0.001)*	−0.039 (−0.077 to −0.001)*

Abbreviations: GA, gestational age; IQR, interquartile range; ln-unit = natural log unit.

a Represents the change in the end point associated with a unit increase in ln(PFOS) or ln(PFOA), which is equivalent to a 2.7-fold increase in PFOS or PFOA.

b Interquartile range is 3.4–7.9 ng/mL for PFOS and 1.2–2.1 ng/mL for PFOA.

c Adjusted for maternal age, BMI, race, previous preterm birth, smoking, diabetes, and hypertension.

d Adjusted for gestational age, maternal age, BMI, race, parity, smoking, baby sex, height, net weight gain, diabetes, and hypertension. For head circumference, adjusted model also includes delivery mode (C-section/vaginal).

* Statistically significant (*p* < 0.05).
